# Instrumentation Failure in Adult Spinal Deformity Patients

**DOI:** 10.3390/jcm13154326

**Published:** 2024-07-24

**Authors:** David P. Falk, Ravi Agrawal, Bijan Dehghani, Rohit Bhan, Sachin Gupta, Munish C. Gupta

**Affiliations:** 1 Department of Orthopaedic Surgery, Washington University in St. Louis, 660 South Euclid Ave, MSC 8233-04-05, St. Louis, MO 63110, USAmunishgupta@wustl.edu (M.C.G.); 2 Hospital of the University of Pennsylvania, Department of Orthopaedic Surgery, 3737 Market Street, Philadelphia, PA 19104, USA

**Keywords:** spinal deformity, instrumentation failure, rod fracture, proximal junctional kyphosis, proximal junctional failure

## Abstract

In recent years, advances in the surgical treatment of adult spinal deformity (ASD) have led to improved outcomes. Although these advances have helped drive the development of deformity surgery to meet the rising volume of patients seeking surgical treatment, many challenges have yet to be solved. Instrumentation failure remains one of the most common major complications following deformity surgery, associated with significant morbidity due to elevated re-operation rates among those experiencing mechanical complications. The two most frequently encountered subtypes of instrumentation failure are rod fracture (RF) and proximal junctional kyphosis/proximal junctional failure (PJK/PJF). While RF and PJK/PJF are both modes of instrumentation failure, they are two distinct entities with different clinical implications and treatment strategies. Considering that RF and PJK/PJF continue to represent a major challenge for patients with ASD and deformity surgeons alike, this review aims to discuss the incidence, risk factors, clinical impact, treatment strategies, preventive measures, and future research directions for each of these substantial complications.

## 1. Introduction

With life expectancy on the rise and fertility rates declining, the portion of the global population aged over 65 continues to expand [1]. This epidemiological shift toward a more elderly society has resulted in an increased prevalence of chronic diseases as well as degenerative musculoskeletal conditions, including adult spinal deformity (ASD) [2,3].

ASD represents a broad spectrum of abnormalities primarily affecting the thoracolumbar spine, resulting in abnormal alignment in the sagittal and/or coronal planes [1,4]. The two most common underlying causes of ASD are iatrogenic flat back and progressive degenerative scoliosis [1]. Additional etiologies leading to ASD include pre-existing adolescent idiopathic scoliosis persisting and progressing in adulthood, progressive kyphosis, and post-traumatic deformity. Although ASD encompasses a broad disease category that may stem from multiple distinct etiologies, it preferentially afflicts the elderly population, with a prevalence of 68% compared to the 32% prevalence estimated for the general population [5]. Given the high prevalence of ASD among the elderly, combined with the progressive aging of the global population, clinicians worldwide will be faced with increasing volumes of elderly patients seeking treatment for ASD. 

Within this group of patients, many may be successfully treated with non-operative measures such as education with routine follow-up, physical therapy, oral medications, or targeted injections [4]. However, if these conservative measures fail, or patients develop neurological symptoms, disability, severe pain, or curve progression, surgery may be considered. 

Although surgical treatment of ASD is considered to be associated with an elevated risk profile, with some studies citing up to a 70% complication rate, it is often warranted given the severe and detrimental impact ASD may have on quality-of-life measures [6]. ASD patients have been shown to have lower SF-36 scores in all domains when compared to patients with other chronic diseases including congestive heart failure, chronic lung disease, and diabetes [7]. Similarly, others have demonstrated that patients with severe sagittal malalignment and lumbar scoliosis have SF-36 Physical Function Scores lower than patients who have limited use of their arms/legs [8]. With surgical treatment, however, patients with ASD have been shown to experience significant improvements in many patient-reported outcome measures, with the most significant improvements being seen in those with more severe ASD pre-operatively [9,10].

Surgical treatment for ASD falls into several different categories depending on the flexibility, location, and magnitude of the deformity. It most commonly involves posterior instrumented fusion of the lumbar or thoracolumbar spine in association with the decompression of associated spinal stenosis [11]. Anterior release, anterior fusion, osteotomies of varying complexity and invasiveness, and vertebral column resection may also be performed depending on the deformity.

In recent years, the approach to the surgical treatment of ASD has evolved in an effort to reduce morbidity and improve outcomes. The use of standardized pre-operative radiographic measurements and three-dimensional modeling has improved surgical planning and promoted targeted correction [4]. Technological advances with intra-operative navigation, improved instrumentation, and minimally invasive approaches collectively represent additional enhancements for the field of spinal deformity surgery [4].

Although these advancements have helped drive the development of deformity surgery to meet the rising volume of patients seeking surgical treatment for ASD, many challenges have yet to be solved. Overall complication rates following corrective surgery remain elevated, ranging up to 70% [6,12]. Instrumentation failure has been shown to be one of the most common major post-operative complications, associated with significant morbidity due to elevated re-operation rates among those experiencing mechanical complications [13,14,15,16]. As a category, instrumentation failure encompasses rod fracture (RF), proximal junctional kyphosis (PJK), proximal junctional failure (PJF), distal junctional kyphosis (DJK), screw loosening or breakage, as well as painful/prominent implants. 

Within this complication category, the two most frequently encountered forms of instrumentation failure include RF and PJK/PJF [6,14,15]. While RF and PJK/PJF are both modes of instrumentation failure, they are two distinct entities with different clinical implications and treatment strategies. Considering that RF and PJK/PJF continue to represent a major challenge for patients with ASD and deformity surgeons alike, we performed a comprehensive review across three databases (PubMed, Web of Science, and Google Scholar) to provide an up-to-date review discussing the incidence, risk factors, clinical impact, treatment techniques, preventive measures, and future research directions for each of these substantial complications.

## 2. Part I: Rod Fracture

### 2.1. Overview

RF continues to represent one of the most common reasons for instrumentation failure following surgery for ASD [6]. The mechanism leading to RF is material fatigue, which in itself is influenced by cyclic loading, rod material, contouring, notching, and surface irregularities [17]. It occurs most commonly at the lumbosacral junction, even when multi-rod constructs are utilized, though elevated rates of RF are also commonly seen at the level of pedicle subtraction osteotomy (PSO) [18,19,20]. This is largely thought to be related to the increased mobility of the lumbar spine along with its increased weight-bearing properties relative to the more cranial spinal segments [21]. 

While certain patients may be asymptomatic, those presenting with mechanical pain or loss of deformity correction frequently undergo revision surgery [22,23]. Given its significant burden, RF has received increased attention in the literature in recent years. However, even with research efforts focusing on the topic, RF remains a challenging problem that continues to limit outcomes of ASD surgery. A large reason for this is that prior reports on RF have had significant limitations, including lack of long-term follow-up, single-surgeon/single-center cohorts, mixed populations in terms of deformity subtypes/levels involved, as well as a general lack of granular data [22]. The goal of this section is to summarize existing evidence to provide an update on the current understanding of the incidence, classification, and risk factors for RF. Current trends in treatment and prevention strategies will also be discussed. 

### 2.2. Incidence

An accurate or precise estimate of the incidence of RF is largely limited by the heterogeneity of the reports in the literature. However, within these confines, several estimates as well as important trends have been identified. In a 2012 retrospective multicenter study including 442 patients, Smith et al. found that symptomatic RF occurred in 6.8% of all deformity patients and 15.8% of patients who underwent pedicle subtraction osteotomy (PSO), most commonly within the first year [23]. Beyond providing an early estimate of the overall rate of RF using a multicenter model, the authors also identified patients undergoing PSO as a subpopulation of ASD patients in whom rates of RF are exceedingly high. Both the overall rate of RF and rates of RF in patients undergoing PSO were found to be even higher in a 2014 prospective multicenter study by Smith, climbing to 9.0% and 22.0%, respectively. More recently, a 2021 meta-analysis of seven studies (including the two previously mentioned) on patients with ASD who underwent surgery and had at least 1 year of follow-up, Noh et al. found an overall incidence of RF of 12% at a mean of 23.2 months after surgery [24]. While this pooled overall estimate is nearly twice the 6.8% originally reported by Smith et al. in the 2012 landmark study, more recent reports with longer follow-up data suggest the true incidence is even higher. In their 2022 multicenter prospective report on 160 patients, Sardi et al. found that 39% of patients had at least 1 rod fracture at a median of 5.1-year follow-up, with RF rates increasing to 49% at 8 years after the index surgery [22]. Collectively, the literature has demonstrated that with increasing duration of follow-up, the overall incidence of rod fracture among all patients continues to rise, highlighting the need for longer-term follow-up in generating a more accurate estimate of the incidence of RF.

### 2.3. Rod Fracture Classification

Not all rod fractures are created equally. Though radiographic RF may be a harbinger of pseudarthrosis in patients with progressive pain, loss of correction, or neurologic deterioration, it is not uniformly a cause for clinical concern, particularly in the absence of symptoms. The radiographic evaluation of RF can be performed using a framework assessing the number of rods fractured as well as RF displacement [25]. RF is considered displaced if there is ≥1 mm on the posterior to anterior (PA) radiograph or ≥50% of the rod diameter on the lateral image [25]. 

In a 2020 retrospective single-center cohort study of 526 patients in whom 96 developed rod fracture, Lertudomphonwanit et al. found several differences between patients with unilateral rod fractures compared to patients with bilateral rod fractures. Whereas those who were asymptomatic with unilateral RF maintained alignment parameters and functional outcome measure improvements, patients with bilateral RF radiographically experienced a loss of sagittal deformity correction and clinically experienced worsening functional outcomes, more frequently resulting in revision than their unilateral RF counterparts (75% vs. 21%, *p* < 0.0001) [26]. Similar results have been reported in the literature, with some studies reporting 100% revision rates in the setting of bilateral RF as compared to under 5% with unilateral RF [20].

Displacement was also found to be associated with revision, with unilateral displaced RF requiring revision more commonly that unilateral non-displaced RF [20,25]. This study highlights the importance of classifying RF and provides surgeons with evidence that can be used to counsel patients when RF is encountered radiographically. While an asymptomatic unilateral non-displaced RF in isolation is unlikely to affect global balance or functional outcome measurements, bilateral displaced fractures commonly signal pseudarthrosis and may require subsequent revision surgery. However, given that with longer term follow-up, patients with unilateral RF may develop bilateral RF, up to 35% in some series, additional clinical and radiographic follow-up is still warranted [22]. Lastly, although there is a theoretical risk of fretting wear and metallosis if broken rods are observed rather than surgically stabilized, very few cases have been reported in the literature [27]. 

### 2.4. Risk Factors

Risk factors contributing to RF can be evaluated in the context of several distinct categories, broken down into demographic, radiologic, and surgical risk factors. Among the demographic risk factors, advanced age, higher body mass index (BMI), and history of prior spine surgery were found to be significantly associated with increased rates of RF in a 2021 meta-analysis published by Noh et al. [24]. Although earlier reports, including a 2014 multicenter prospective study, have also cited female sex, osteoporosis, and tobacco use as demographic risk factors for RF, no significant association was found in the meta-analysis previously described [19,24]. More recently, Sardi et al. failed to identify any demographic risk factors for RF in their 2022 multicenter prospective study, noting that age, BMI, osteoporosis, and tobacco use were not significantly associated with RF [22]. Ultimately, demographic risk factors for rod fracture vary throughout the literature, indicating that further research on this subset of risk factors is warranted.

Similar to the demographic risk factors, radiographic risk factors for RF vary depending on the study. Radiographic risk factors were also assessed in the meta-analysis previously mentioned by Noh et al., which noted significantly higher rates of RF associated with larger pre-operative pelvic tilt (PT) and thoracic kyphosis (TK) [24]. Although several of the studies included in the meta-analysis found higher pre-operative sagittal vertical axis (SVA) to be a risk factor for RF, no significant association between SVA and RF was found in their statistical analysis [19,24,26]. Other subsequent studies have identified higher pre-operative lumbar lordosis (LL) to be associated with RF, finding a mean LL of 39.5° in patients who sustained RF compared to 34.0° in those who did not (*p* = 0.03) [22]. One possible explanation for this finding could be related to increased rod contouring required in patients with higher pre-operative LL, meaning that patients with increased lumbar lordosis at baseline require more bending to appropriately contour the rod, thereby potentially weakening the rod and increasing the risk of RF [21]. Post-operative radiographic measurements that have been associated with rod fracture include higher PI-LL mismatch and post-operative PT [22]. 

Whereas the literature varies in terms of demographic and radiographic risk factors for RF, there is more consistency and agreement surrounding some of the surgical risk factors for RF. One of the most reported surgical risk factors associated with RF is PSO [19,23,24]. In one of the largest studies on instrumentation failure in spinal deformity, Smith et al. found an RF rate of 22% in patients who underwent PSO, compared to an RF rate of 4.7% in cases that did not include PSO [19]. Similarly, PSO was found to be significantly associated with higher rates of RF in the recent meta-analysis by Noh et al. [24]. This finding can be attributed to the increased biomechanical stress placed upon the rods, given that a PSO is inherently a destabilizing procedure, requiring removal of the posterior elements, bilateral pedicles, and adjacent facets. Therefore, the rods are required to do more work to maintain the correction. In addition, the rods themselves may be weakened by the significant contouring required to maximize the amount of deformity correction achieved through the PSO. A biomechanical study by Tang et al. emphasizes this concept, in which the authors demonstrated that increasing rod angular contour lowers the fatigue life of the rods, leading to weaker constructs [21]. Strategies to mitigate the stress on the rod in cases including PSO, such as the use of multiple rod constructs, will be discussed later in this section.

In addition to pedicle subtraction osteotomy, several other surgical factors have been identified as risk factors for RF. In a retrospective single-center cohort study of 526 patients who underwent spinal deformity surgery, an increased number of vertebrae fused for patients who received less than 12 mg rhBMP2 per level, and the use of 5.5 mm cobalt chromium rods was found to have a significant association with increased rates of RF [26]. Estimated blood loss (EBL) has also been shown to be a surgical variable associated with RF, with higher EBL potentially indicating more case complexity and invasiveness [22]. 

### 2.5. Treatment Strategies

Given that RF remains a challenge in spinal deformity, surgeons must be familiar with the different treatment modalities for this complication. Ideally, the best treatment strategy regarding RF is prevention. However, the approach to the treatment of RF depends upon shared decision-making between the treating surgeon and patient regarding the patient’s symptoms, physical examination, and willingness and ability to tolerate further surgery. While asymptomatic patients with unilateral, non-displaced RF may be counseled and observed with repeat clinical and radiographic follow-up, most patients with RF report significant lower back pain and are candidates for surgical intervention [28]. 

**Figure 1:**
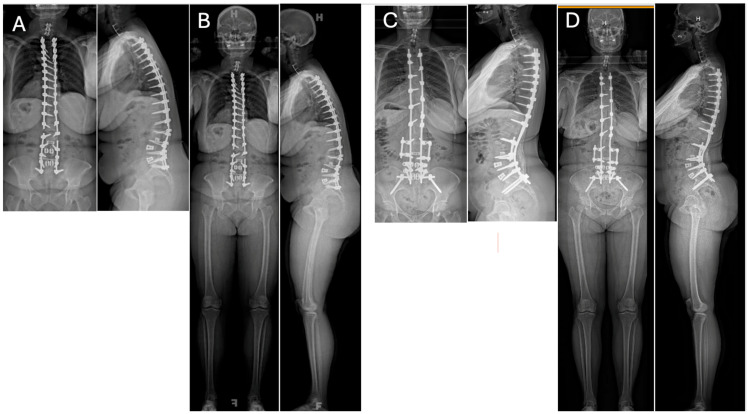
36″ (**A**) and EOS (**B**) Postero-Anterior (PA) and lateral radiographs of a 49-year-old female who had a previous posterior spinal fusion from T3 to the pelvis which failed with broken instrumentation and pseudoarthrosis. The patient fell forward in the sagittal plane resulting in sagittal decompensation and malalignment causing debilitating pain. 36″ (**C**) and EOS (**D**) PA and Lateral radiographs 3 years post-operatively from the removal of instrumentation, revision T3-Pelvis posterior spinal fusion with quarter-inch stainless steel rods and pedicle subtraction osteotomy (PSO) at L3 using the satellite rod configuration at the PSO site.

Pre-operatively, full-length standing radiographs should be obtained to evaluate sagittal parameters. A CT scan should be obtained to evaluate for pseudarthrosis or screw loosening not apparent on radiographic imaging. In patients with pseudarthrosis and maintained correction, revision arthrodesis with or without interbody fusion, along with the upsizing of any loose pedicle screws and rod replacement may be considered [23]. In patients with pseudarthrosis and persistent or recurrent sagittal malalignment, additional correction should be obtained with interbody cages and/or posterior-based osteotomies depending on the amount of correction required (Figure 1). Subsequent rod replacement with or without the placement of accessory or satellite rods can then be performed. 

In a recent study evaluating treatment strategies for RF, Yamato et al. reported on 54 RFs in a cohort of 304 total patients undergoing deformity correction and fusion for ASD [28]. Thirty-six patients underwent revision surgery, which involved bilateral rod replacement and satellite rod placement across all lumbar levels, as well as transforaminal interbody fusions (TLIFs) at posterior column osteotomy sites. The remaining 18 patients, most of whom were asymptomatic, were managed successfully with thoracolumbar orthoses and had no evidence of significant deformity progression at a mean follow-up of 18.5 months after RF development. This study supports the notion that RF can be treated both operatively and conservatively depending on the degree of symptoms.

From the biomechanical perspective, a different in vitro study compared construct stiffness amongst various ‘direct-repair’ strategies for RF such as in-line rod connectors to bridge the fracture gap, rod couplers, and accessory rods spanning the fracture [29]. The authors found that the usage of accessory rods and crosslinks in combination provided the most augmented stability to flexion-extension, lateral bending, and axial rotation stress testing. 

Overall, treatment for RF remains dependent upon the patient’s specific case and the treating surgeon’s experience in managing these issues. The above described strategies represent attempts to increase construct stiffness and provide more rigid biomechanical stability to multiplanar forces to treat RF in revision surgery. 

### 2.6. Prevention Strategies

Several strategies have been proposed to reduce the rates of RF in spinal deformity surgery. Broadly, these methods aim to increase construct stiffness, decrease rod fatigue failure to prevent later RF, and limit micromotion to facilitate osseous fusion across intended levels. 

In a retrospective review of 178 patents with adult spinal deformity, Lee et al. demonstrated decreased rates of RF with the use of 6.35 cobalt chrome rods, accessory rods, and the use of lateral lumbar interbody fusion (LLIF) [30]. Similarly, Smith et al. also found significantly lower RF rates when cobalt chromium rods (2.7%) were used rather than stainless steel (7.4%). or titanium alloy (8.6%) [23]. The authors also hypothesized that using pre-contoured rods or using plate benders to contour a flat rod could limit the development of intra-operative surface rod irregularities and eventual RF. 

In addition, the use of interbody fusion at the caudal aspect of long spinal deformity constructs have been thought to reduce the development of RF during the primary treatment of ASD. In a study comparing RF rates with TLIF vs. anterior lumbar interbody fusion (ALIF) at the caudal aspect of long spinal deformity constructs, Adogwa et al. demonstrated a lower incidence of bilateral RF in the ALIF group [20]. However, the ultimate revision rate was similar between groups, and the decreased incidence of bilateral RF in the ALIF group was attributed to a higher average amount of rhBMP-2. Given that favorable results have been observed with ALIF, TLIF, and LLIF, the decision on which technique to utilize is primarily a function of surgeon experience along with individual patient parameters. Robust evidence is lacking to assert the superiority of one interbody technique over another.

Beyond the use of stiffer rods, accessory rods, and interbody devices presented, more specific strategies must be considered when performing pedicle subtraction osteotomy. In response to the elevated rates of RF observed in standard 2-rod constructs for patients undergoing PSO, in 2002, a senior author developed a 4-rod technique in which two additional, short, independent “satellite” rods are placed spanning only the level of the PSO [31], separate from the long rods spanning the entire construct. Compared to accessory rods, which are connected to the primary rod, satellite rods are not connected to the primary rod. Advantages afforded by the satellite rods include the following: (1) Controlled closure of the osteotomy site; (2) Avoidance of the need for significant conventional rod contouring and angular bending at the level of the PSO, which is thought to weaken the rod and predispose it to fracture; (3) Greater stability across the osteotomy site with four rods, given the inherent instability and elevated mechanical stresses at this level.

The efficacy of this technique was evaluated in a study comparing the aforementioned 4-rod technique using satellite rods with the conventional 2-rod approach. Amongst 29 patients in the 4-rod cohort, at final follow-up, the RF rate was 0%, and the pseudoarthrosis rate was 3.4, both significantly lower than the RF rate of 25% and pseudarthrosis rate of 25% in the 2-rod group [31]. Similarly, Hyun et al. demonstrated significantly lower rates of pseudarthrosis in patients with 4-rod constructs (15%) compared to 2-rod constructs (29%) who underwent three-column osteotomy [32]. 

Although 4-rod constructs have been shown in multiple studies to lower the rates of RF, pseudarthrosis and RF still occur. This has prompted some surgeons to use “super” multi-rod constructs utilizing 5 or 6 rods. Although the optimal number of rods as well as the configuration of the additional accessory or satellite rods remains a question, from a biomechanical perspective, adding accessory rods to create a 5- or 6-rod construct has been shown to increase construct rigidity, leading to decreased global range of motion (ROM) and local ROM at the PSO site, and decreased rod stress relative to 2- and 4-rod constructs [33]. However, the increased stiffness of the construct posteriorly limits load transfer to the anterior column, which may negatively alter the healing properties of the anterior column or delay rod failure.

Clearly, additional clinical research investigating the impact of the number of rods and rod configuration on RF and pseudarthrosis is warranted to improve our understanding of this complex relationship. As surgeons and researchers consider future studies evaluating different multi-rod constructs (MRCs) across three-column osteotomies (3COs), the authors encourage the adoption of the classification system described by El Dafrawy et al. to allow for a structured, standardized comparison [34]. 

### 2.7. Future Directions

Looking ahead, surgeons and industry representatives will continue working toward developing and improving access to pre-operatively manufactured, machine-contoured rods as a means of decreasing RF rates. Not only would machine-bent rods minimize the need for the significant intra-operative angular contouring that can decrease the fatigue strength of rods, but pre-planned rods may better allow surgeons to meet their pre-operative alignment targets [35]. Both have the potential to enhance the fatigue life of the rods, thereby allowing for more time for fusion to occur.

While patient-specific rods are receiving increased attention, particularly with a manufacturer-quoted RF rate of 2.2%, the existing literature describing cohorts treated with patient specific rods is extremely heterogenous in terms of surgical technique, pre-operative diagnosis, rod material, and outcome measures/complications reported [36]. In a systematic review published earlier this year, Picton et al. discuss the results reported by seven studies including 304 patients with regard to experience with patient-specific rods. Only three of the studies reported on RF, with reported RF rates of 0% (0/60), 9% (8/86), and 50% (6/12) [36,37,38,39]. Given the small sample sizes, limited follow-up, and heterogeneity of patients and surgical procedures, it is difficult to draw any robust conclusions comparing patient-specific rods to traditional rods at this moment in time.

Further research efforts should attempt to use standardized methodologies and specific subgroups of patients to directly compare patient-specific rods to traditional rods to better evaluate their impact on RF. Outside of the rods themselves, additional investigation into the optimal number of rods and configuration of MRCs is warranted to enhance our understanding of the ideal indication for the multiple MRC options.

## 3. Part II: Proximal Junctional Kyphosis and Proximal Junctional Failure

### 3.1. Overview

Proximal junctional kyphosis (PJK) describes the radiographic phenomenon for the vertebrae just proximal to the uppermost instrument vertebrae (UIV) to experience kyphotic deformation relative to the UIV [40,41]. This is often a dynamic process that can be acute or progressive following adult and adolescent spinal deformity surgery [42,43,44]. The parameters that define PJK were first described in the literature by Glattes et al. as a proximal junctional angle between the lower endplate of the UIV and the upper endplate of the vertebrae 2 levels cephalad to the UIV (UIV+2) >10° or >10° of the pre-operative angle [45,46]. It most commonly occurs in patients who undergo long construct fusion at the thoracolumbar junction; however, it can be present along any point of the spine [47]. It most commonly occurs due to a failure of the posterior vertebral ligamentous complex and often lacks neurologic symptoms or pain [42,48].

### 3.2. Incidence

While the exact incidence of PJK is debated, the current literature suggesting 20–40% of patients receive spinal fusion surgery for adult spinal deformity, with some reports suggesting an incidence as high as 61.7% [44,49,50,51,52]. In two separate meta-analyses performed by Liu et al. and Luo et al., they found the incidence of PJK to be 30% and 32.2%, respectively [53,54]. Furthermore, in a 2014 survey of 226 surgeons at the scoliosis research society meeting, nearly a quarter (24.8%) of the respondents reported seeing PJK in 21% or more of the patients. Additionally, they found that PJK was listed as one of the top 5 indications for revision surgery [55].

### 3.3. Classification

Classifications for PJK have been proposed in an attempt to create a standardized language to describe and communicate the severity of PJK/PJF, however, with limited clinical utility. Yagi et al. created a classification system that provides a description of the severity of PJK that is simple and reproducible (Table 1). The classification is divided into Type 1, 2, and 3, where Type 1 = ligamentous failure, Type 2 = bone failure, Type 3 = bone or implant failure; Grade A, B, and C corresponding to increase in kyphotic angle—10° to 14°, 15° to 19°, or >20°, respectively; and lastly, the presence (S) or absence (N) of spondylolisthesis [44,56]. More recently, Hart et al. and the International Spine Study Group (ISSG) created the PJK severity scale which relies on six parameters: neurologic deficit, focal pain, instrumentation problem, change in kyphosis/posterior ligamentous complex integrity, UIV/UIV+1 fracture, and the level of UIV (Table 2) [57].

Despite the relatively high incidence of PJK, the majority of patients are pain-free and without neurologic symptoms [42]. However, the spectrum of PJK is wide, with the literature supporting the association of greater kyphotic angles with worse pain scores and the presence of upper back pain as a predictor of PJK [44,58]. At the extreme of the PJK spectrum, patients experience both kyphosis as well as a failure of the structural components, including the posterior vertebral ligamentous complex and/or the vertebral body; this occurrence is termed proximal junctional failure (PJF). Although the exact definition of PJF remains controversial, the clear distinction lies in the associated structure failure that almost always necessitates surgical intervention [59].

Many view PJF as an extreme on the spectrum of PJK; Yagi et al. described PJF as any symptomatic PJK requiring operative intervention [56]. Taking it a step further, Hastens et al. described PJF as 15° or more of PJK along with a fracture of the UIV or UIV+1, failure of UIV fixation, or need for extension of instrumentation within 6 months of the index surgery [60]. Lastly, Hart et al. described PJF as a 10° or greater post-operative increase in kyphosis between the UIV and UIV+2, along with one or more of the following features: a fracture of the vertebral body of the UIV or UIV+1, posterior osseo-ligamentous disruption, or pullout of instrumentation at the UIV [61].

### 3.4. Risk Factors

Regardless of the definition used, PJF and the resulting need for revision surgery independently predict poor outcomes following surgery for ASD [49]. For this reason, the recent literature has focused on identifying predictive measures of PJK and PJF including osteoporosis, higher body mass index (BMI), age over 55 years at the time of index surgery, thoracic kyphosis >40°, sagittal vertebral axis (SVA) greater than 5cm, degeneration of paravertebral muscles, over-correction of lumbar lordosis, hybrid fixation, patients who receive anterior and posterior approach, fixed fusion to the pelvis, and lack of a ligament reinforcement device at UIV and proximal vertebrae in the thoracolumbar junction [52,53].

### 3.5. Treatment Strategies

The treatment of PJK and PJF remains a significant challenge facing surgeons. PJK may be asymptomatic and require only observation [45]. However, PJF is typically accompanied by symptoms of pain, deformity, and imbalance and requires more than conservative management (pain management, brace, and physical therapy) [56]. Multiple treatment strategies have been discussed in the literature, including the optimization of bone health, revision surgery with extension of fusion to proximal levels, osteotomies or vertebral column resection (VCR) as needed for further sagittal balance correction, the reinforcement of UIV and UIV+1, and possible anterior column support [62]. Importantly, a thorough pre-operative discussion with patients suffering from PJF must touch on the high risk of recurrent PJF despite revision surgery for PJF itself [60,63,64,65,66,67,68,69]. 

Medically, osteoporosis and other metabolic bone conditions must be identified pre-operatively and managed aggressively [63]. Treatment in this regard typically occurs via consultation with a specialized endocrinologist. Perioperative bone metabolic protocols for revision surgery vary in the length of medication usage and type but typically include teriparatide and/or bisphosphonates. Left unchecked, osteopenia and poor bone quality hinder a surgeon’s ability to achieve rigid spinal fixation due to decreased screw-pullout strength. These patients may be predisposed to proximal junctional fractures that lead to PJK and eventual PJF [64]. 

The surgical intervention strategy should be tailored to each individual patient’s symptoms and pathology as elucidated by detailed physical examination and advanced imaging work-up, which typically includes full-length standing films, CT, and MRI. The pre-operative selection of a more proximal UIV is critical to bypass the area of focal kyphosis and achieve adequate cranial fixation [60]. Unfortunately, there is no consensus on appropriate UIV selection in the revision setting for PJK/PJF, and it remains dependent upon the treating surgeon’s preferences and experiences. Intra-operatively, the surgical approach may be carried out via an anterior, posterior, or combined approach to achieve desired deformity correction and possibly neural decompression if indicated. An anterior approach in the revision surgery can be ideal if additional support is desired to treat vertebral collapse or fracture via interbody fusion [65]. 

**Figure 2:**
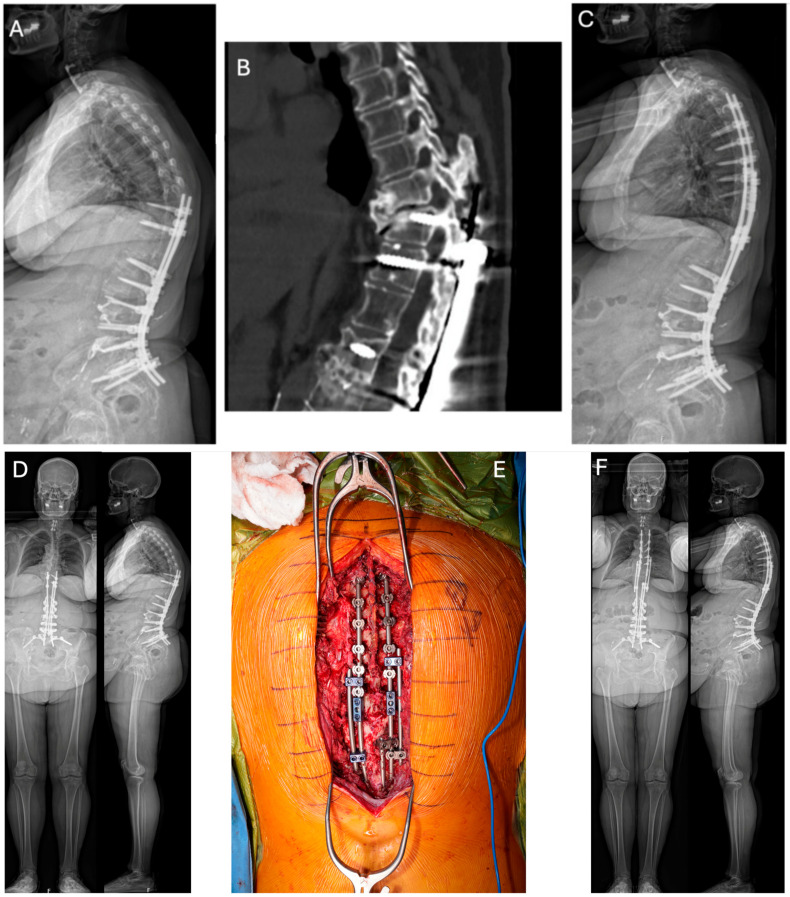
Lateral 36″ radiographs (**A**,**C**) and sagittal CT reconstruction (**B**) of a 58 year-old female with history of combined L1–L5 anterior lumbar interbody fusion and T10-Pelvis posterior spinal fusion, complicated by infection requiring irrigation and drainage, who presented 1 year post-operatively with proximal junctional kyphosis (**A**,**B**). She underwent removal of instrumentation, revision posterior spinal fusion from T4 to T11 with Smith-Peterson osteotomy at T9-T10 (**C**). (**D**) Pre-operative EOS PA and lateral radiographs demonstrating proximal junctional kyphosis with global sagittal imbalance in the same patient shown in (**A**,**B**). (**E**) (top = cranial; bottom = caudal): Intra-operative clinical photo demonstrating the final construct used to address the proximal junctional failure and restore the sagittal plane following Smith-Peterson osteotomy at T9–T10. End-to-end connectors were utilized to connect the rods placed in the new instrumentation from T4 to T9 with the previously placed instrumentation. Side-to-side connectors were also placed at T8–9 on the left and T7–8 on the right and connected at T12 on both sides. (**F**) Post-operative EOS PA and lateral radiographs highlighting improved global alignment following removal of instrumentation, revision posterior spinal fusion from T4 to T11 with Smith-Peterson osteotomy at T9–T10.

Once the spine is re-exposed, primary instrumentation is typically partially or wholly removed, and the fusion mass is inspected for any defects requiring further bone grafting, decortication, or augmentation with biologic agents such as BMP [66]. Smith-Peterson osteotomies, PSO, or VCR may be performed to correct the focal PJK leading to instrumentation failure (Figure 2A,B), as residual kyphosis poses a significant risk factor for PJK/PJF recurrence [67]. The augmentation of fixation at the UIV to prevent recurrent PJF has also been heavily explored. This has historically involved large diameter UIV pedicle screws of maximum length coupled with polymethylmethacrylate (PMMA) cement augmentation to decrease screw pullout and the risk of construct failure [68,69]. Sublaminar tethering to augment the posterior ligamentous tension band cranially in the revision setting has also been explored but is primarily a prevention strategy [70]. A combination of medical optimization and thoughtful surgical technique are critical to achieving a successful result when treating PJK/PJF. 

### 3.6. Prevention Strategies

Given that the exact pathogenesis of PJK/PJF is not fully understood, in part due to the multiple variables thought to contribute to the complication, it cannot be prevented entirely. However, several strategies have been proposed and evaluated in the literature to limit the risk of developing PJK/PJF. These include both pre-operative considerations such as patient selection and level selection, along with intra-operative techniques including avoiding over-correction and utilizing either hooks, cement augmentation, or tethers at the proximal junction [62]. Tapered rods and multi-material constructs have also been proposed as potential intra-operative techniques to reduce the rates of PJK/PJF, though further evaluation of these concepts is ongoing [71].

Pre-operative prevention begins with patient selection. Comorbidities and modifiable risk factors should be addressed and optimized prior to surgery. Chief among these is osteoporosis. 

Pharmacologic treatment with medications to increase formation or decrease bone resorption are critical in improving bone mineral density. Anabolic agents stimulating bone formation include teriparatide (recombinant parathyroid hormone), abaloparatide, as well as the monoclonal antibody romosozumab [62]. Medications that limit bone resorption include denosumab, which is a monoclonal antibody to RANKL, as well as bisphosphonates. Of these, perioperative teriparatide has been studied in the context of PJK by Yagi et al. The authors demonstrated improved bone mineral density at the UIV and UIV+1 compared to controls after 6 months of treatment, with a lower incidence of PJK at two years [64]. Although these results were both intuitive and encouraging, a more recent study by Mohanty et al. failed to show a significant difference in PJK rates in their comparative study of ASD patients with osteoporosis treated with teriparatide vs. osteopenic patients who did not receive teriparatide [72]. However, the lack of a difference is likely attributed to the small number of patients in each group who developed PJK, given that only 6/78 in the group treated with teriparatide vs. 10/156 in the osteopenia group developed PJK 2 years post-operatively. Ultimately, the choice of medical therapy directed at improving bone mineral density depends on individual patient factors, and the decision should incorporate a bone health expert.

Once a patient has been optimized, a pre-operative plan including the selection of the UIV is determined. Broadly, the construct may end in either the distal thoracic spine (T9–L1) or the proximal thoracic spine (T2–T5). While the indications to extend to the proximal thoracic spine are well established, including structural scoliosis, thoracic hyper-kyphosis, and thoracolumbar junctional kyphosis, the impact of this decision on PJK rates has been less well-reported. In a retrospective comparative study on 89 ASD patients with 2 years of follow-up, Ha et al. found no significant difference in the incidence of PJK between the 67 patients in whom the UIV was in the distal thoracic spine as compared to the 22 patients in whom the UIV was in the proximal thoracic spine [73]. However, the types of PJK were different, with compression fracture occurring more commonly in the distal thoracic group, whereas subluxation was more common in the proximal thoracic group. Ultimately, however, both groups achieved improvements in segmental/global sagittal balance as well as quality of life measures. Within the distal thoracic spine itself, others have demonstrated that a UIV of T10 has a significantly higher risk of PJK when compared to adjacent vertebrae of T9 or T11 [74].

In addition to the pre-operative considerations related to patient selection/optimization and level selection, several surgical strategies and techniques have been described in an effort to reduce the rates of PJK/PJF. From a global balance perspective, the goal is to correct sagittal malalignment such that the head is harmoniously balanced over the femoral heads. The rates of PJK have been found to be elevated in patients who are sagittally over-corrected [75]. More specifically, patients with PJK and an upper thoracic UIV had an under-correction of thoracic kyphosis, whereas patients with PJK and a distal thoracic UIV tended to have an over-correction of lumbar lordosis [75]. 

Beyond harmoniously balancing the spine to reduce PJK/PJF, several surgical techniques have been described to address the proximal junction itself to limit failure. The use of transverse process (TP) hooks at the UIV has been described to create a softer transition between the rigid instrumented construct and the mobile, un-instrumented spine above. In their retrospective review comparing TP hooks to pedicle screws in adults undergoing long fusion, Hassanzadeh et al. found 0% of patients with TP hooks at the UIV developed PJK compared to nearly 30% of those with pedicle screws at the UIV [76]. 

Another technique that can be considered at the proximal junction is ligament augmentation with polyethylene tape looped through or around the spinous process of the UIV, UIV+1, and/or UIV+2. This technique is thought to work by reinforcing the posterior tension band (consisting of supraspinous and interspinous ligaments) to decrease junctional stresses while simultaneously strengthening the UIV and adjacent segments [77]. Safaee et al. found significantly lower rates of re-operation for PJF in their analysis of 242 ASD patients treated with ligament augmentation compared to 77 treated without (3.3% vs. 15.6%, *p* < 0.001) [77]. Biomechanical studies evaluating the ideal tether configurations have supported going to the UIV+2 with the tether looped around the UIV and UIV+2 or woven through UIV, UIV+1, and UIV+2, as these configurations best decreased the junctional range of motion and adjacent segment stress [78].

The proximal junction can also be stabilized with vertebroplasty using polymethylmethacrylate (PMMA) cement at the UIV and UIV+1 [79]. In a biomechanical study using a cadaveric model, Kebaish et al. showed that a prophylactic vertebroplasty of the UIV and UIV+1 decreased the rates of junctional fractures following long spinal instrumentation, when compared to UIV vertebroplasty or no vertebroplasty [79]. This approach has significant value, particularly in patients with poor bone quality, as cement augmentation has been shown to increase screw-pullout strength compared to uncemented screws by stabilizing the screw–bone interface [80]. Although effective, cement augmentation has several noteworthy disadvantages, including cement embolization into the vascular system, leakage into the spinal canal, and difficulty with removal if subsequent revision is required. 

Whereas TP hooks, ligament augmentation, and vertebroplasty focus entirely on the junctional level, tapered rods and multi-material constructs represent a different approach to reducing the rates of PJK/PJF. This category of PJK/PJK prevention techniques aims to create a rigidity gradient with a more gradual transition in stiffness spread over the entire length of the construct, with progressively decreasing rigidity going from caudal to cranial. Transition rods vary in diameter, and therefore strength, along the length of the singular rod, conceptually allowing for a buffer zone of intermediate stiffness between the stiff caudal end of the construct and mobile un-instrumented spine above [71]. Similarly, multi-material constructs are conceptualized to create a gradient of rigidity that decreases moving cranially, though these constructs include multiple, overlapping rods of differing materials and diameters as opposed to a singular transition rod that, in itself, tapers in diameter [81]. 

While each of the previously mentioned techniques may be used in isolation, they can also be combined. For example, the authors favored an approach to the proximal junction involving TP hooks to create a soft landing while also utilizing a polyethylene tether for ligament augmentation. 

### 3.7. Future Directions

In the coming years, there is immense potential for artificial intelligence (AI) to improve our understanding of PJK/PJF through the efficient analysis of large datasets. AI may be leveraged not only to develop predictive models to identify patients at elevated risk for PJK/PJF pre-operatively, but also to guide surgical decision-making [62]. Further, larger prospective clinical trials comparing PJK/PJF prevention strategies are warranted and will come as our understanding of PJK/PJF continues to improve and longer follow-up data become available.

## 4. Conclusions

Given the aging world population combined with the elevated prevalence of ASD among the elderly, surgeons worldwide will face increasing volumes of patients seeking treatment for ASD. Despite the many advances in terms of pre-operative planning software, minimally invasive approaches, and new technologies like navigation, robotics, and patient-specific instrumentation, mechanical complications following the surgical treatment of ASD persist. This review discusses our current and evolving understanding of rod fracture and proximal junctional kyphosis/failure, the two most common mechanical complications in deformity surgery. 

Importantly, our analysis highlights that it is still difficult to make broad, all-encompassing conclusions regarding the “best” way to prevent or treat RF and PJK. The heterogeneity in patients considered to have ASD (i.e., etiology of deformity, length of fusion constructs, surgical techniques, etc.), combined with a lack of large, randomized studies, limited availability of long-term follow-up, and a preponderance of single-surgeon cohorts, contribute to the fact that RF and PJK remain challenging complications despite the many developments within the field. 

Within these limitations, the authors favor the utilization of the four-rod construct described earlier in the text when performing PSO to limit RF rates in this challenging subpopulation. In terms of PJK/PJF, the combination of transverse process hooks with ligament augmentation using polyethylene tape at the proximal junction is the preferred technique of the senior author for PJK/PJF prevention.

Ultimately, additional research into both topics is required to further develop our understanding of these complications. While randomized, prospective trials comparing specific subsets of ASD patients or surgical techniques certainly represent the gold standard for enhancing our knowledge on the topic, another potential avenue to address current shortcomings in the literature may involve harnessing AI to capture, evaluate, and sort large datasets on specific subsets of ASD patients. Though yet to be demonstrated, it could prove critical in taking deformity surgery to another level, with the goal of limiting mechanical complications to optimize patient outcomes.

## Figures and Tables

**Table 1:** The Boachie-Adjei classification of proximal junctional kyphosis (PJK)/proximal junctional failure (PJF).

Category		Description
Type	Type 1	Disc and ligamentous failure
	Type 2	Bone Failure
	Type 3	Implant/Bone failure
Grade	Grade A	Proximal junction increase of 10–19°
	Grade B	Proximal junction increase of 20–29°
	Grade C	Proximal junction increase ≥30°
Spondylolisthesis	PJF-N	No spondylolisthesis present above the uppermost instrumented vertebra
	PJF-S	Spondylolisthesis present above the uppermost instrumented vertebra

**Table 2:** The Hart-International Spine Study Group description for proximal junctional kyphosis (PJK).

	Characteristic	Severity Score
Neurological Defict:	None	0
	Radicular Pain	2
	Myelopathy or Motor Deficit	4
Focal Pain	None	0
	VAS ≤ 4	1
	VAS ≥ 5	3
Instrumentation Problem	None	0
	Partial Fixation Loss	1
	Prominence	1
	Complete Fixation Loss	2
Change in Kyphosis	0–10°	0
	10–20°	1
	>20°	2
	PLC Failure	2
Upper Instrumented Vertebra Changes	None	0
	Compression Fracture	1
	Burst/Chance Fracture	2
	Translation	3
Level of PJK	Thoracolumbar Junction	0
	Upper Thoracic	1
